# Two *S*-type *Z*-eigenvalue inclusion sets for tensors

**DOI:** 10.1186/s13660-017-1428-6

**Published:** 2017-06-27

**Authors:** Yanan Wang, Gang Wang

**Affiliations:** School of Management Science, Qufu Normal University, Rizhao, Shandong 276826 P.R. China

**Keywords:** 15A18, 15A69, *Z*-eigenvalue inclusion sets, weakly symmetric nonnegative tensors, largest *Z*-eigenvalue

## Abstract

In this paper, we present two *S*-type *Z*-eigenvalue inclusion sets involved with a nonempty proper subset *S* of *N* for general tensors. It is shown that the new sets are tighter than those provided by Wang *et al.* (Discrete Contin. Dyn. Syst., Ser. B 22(1):187-198, 2017). Furthermore, we obtain upper bounds for the spectral radius of weakly symmetric nonnegative tensors, which are sharper than existing results.

## Introduction

Let $\mathcal{C}$ ($\mathcal{R}$) be the set of all complex (real) numbers and $N=\{1,2,\ldots,n\}$. A real *m*-order *n*-dimensional tensor $\mathcal{A}$ consists of $n^{m}$ elements:

$$ \mathcal{A}=(a_{{i_{1}}{i_{2}}\ldots{i_{m}}}),\quad\forall a_{{i_{1}}{i_{2}}\ldots {i_{m}}}\in\mathcal{R}, i_{1}, i_{2}, \ldots, i_{m}\in N. $$

$\mathcal{A}$ is called nonnegative (positive) if $a_{{i_{1}}{i_{2}}\ldots {i_{m}}}\geq0$ ($a_{{i_{1}}{i_{2}}\ldots{i_{m}}}> 0$).

The following two definitions of eigenpairs were introduced by Qi [2] and Lim [3], respectively.

### Definition 1

Let $\mathcal{A}$ be a tensor with order *m* and dimension *n*. If there exist a nonzero vector $x=[x_{1}, x_{2}, \ldots, x_{n}]^{T} \in\mathcal {C}^{n}$ and a number $\lambda\in\mathcal{C}$ satisfying the equation

$$ \mathcal{A}x^{m-1}=\lambda x^{[m-1]}, $$

then $(\lambda, x)$ is called an eigenvalue-eigenvector of $\mathcal {A}$, where

$$ \mathcal{A} x^{m-1}= \Biggl(\sum^{n}_{i_{2}, \ldots, i_{m}=1}a_{{i}{i_{2}}\ldots {i_{m}}}x_{i_{2}} \cdots x_{i_{m}} \Biggr)_{1\leq i\leq n} $$

and $x^{[m-1]}=[x^{m-1}_{1},x^{m-1}_{2},\ldots,x^{m-1}_{n}]^{T}$. $(\lambda,x)$ is called an *H*-eigenpair of $\mathcal{A}$ if they are all real.

### Definition 2

Let $\mathcal{A}$ be a tensor with order *m* and dimension *n*. We say that $(\lambda,x)\in\mathcal{C}\times(\mathcal{C}^{n}\setminus\{0\})$ is an *E*-eigenpair of $\mathcal{A}$ if

$$ \mathcal{A}x^{m-1}=\lambda x \quad\mbox{and}\quad x^{T}x=1. $$

$(\lambda,x)$ is called a *Z*-eigenpair if they are real.

As we know, the *Z*-eigenpair for nonnegative tensors plays an important role in some applications such as high order Markov chains [4, 5] and best rank-one approximations in statistical data analysis [6, 7]. Some effective algorithms for finding *Z*-eigenvalue and the corresponding eigenvector of tensors have been implemented [8, 9]. Generally, we cannot judge that *Z*-eigenvalues generated by the above algorithms are the largest *Z*-eigenvalues. Therefore, the following definitions were introduced and used by Qi [2] and by Chang [8] for studying important characterizations of the largest *Z*-eigenvalue of a tensor.

### Definition 3

[2]

Let $\mathcal{A}$ be a tensor with order *m* and dimension *n*. We define $\sigma(\mathcal{A})$ the *Z*-spectrum of $\mathcal{A}$ by the set of all *Z*-eigenvalues of $\mathcal{A}$. Assume $\sigma(\mathcal{A})\neq \emptyset$. Then the *Z*-spectral radius of $\mathcal{A}$ is denoted as

$$ \rho(\mathcal{A})=\max \bigl\{ |\lambda|: \lambda\in\sigma (\mathcal{A}) \bigr\} . $$

### Definition 4

[8]

Let $\mathcal{A}$ be a tensor with order *m* and dimension *n*. $\mathcal{A}$ is weakly symmetric if the associated homogeneous polynomial $\mathcal{A}x^{m}$ satisfies

$$ \nabla\mathcal{A}x^{m}=m \mathcal{A}x^{m-1}. $$

Based on the weakly symmetric condition, Chang *et al*. [8] established the equivalent relation between the largest *Z*-eigenvalue and *Z*-spectral radius of nonnegative tensors. On the basis of the relationship between the Gelfand formula and the spectral radius, Song *et al*. [10] gave the *Z*-spectral radius bound for nonnegative tensors. He *et al.* [11–13] presented the largest *Z*-eigenvalue for weakly symmetric nonnegative (positive) tensors. Furthermore, Li *et al*. [14] improved some bounds for the eigenvector and *Z*-spectral radius. For general tensors, Wang *et al*. [1] established Gershgorin-type *Z*-eigenvalue inclusion theorems. Moreover, Zhao *et al.* [15] extended some results of [1]. Very recently, Li *et al*. [16] introduced an *S*-partition method and established *S*-type *H*-eigenvalue localization sets, which may reduce computations. Therefore, we want to use the *S*-partition method and propose *S*-type *Z*-eigenvalue inclusion sets for general tensors.

The remainder of this paper is organized as follows. In Section 2, we establish *S*-type *Z*-eigenvalue inclusion sets for general tensors by breaking *N* into a disjoint subset *S* and its complement, which is proved to be tighter than the sets in [1]. In Section 3, as applications of the above results, we propose some new bounds on the *Z*-spectral radius of a weakly symmetric tensor and show that they are tighter than the existing bounds in [1, 8, 10–12, 14] by Example 2.

## *S*-Type *Z*-eigenvalue inclusion sets

In this section, we give *S*-type *Z*-eigenvalue inclusion sets of the tensor $\mathcal{A}$ by dividing *N* into disjoint subsets *S* and *S̄*, where *S̄* is the complement of *S* in *N*. Furthermore, we establish comparisons among different *Z*-eigenvalue inclusion sets.

In what follows, we introduce a lemma for a general tensor.

### Lemma 1

Theorem 3.1 of [1]

*Let* $\mathcal{A}$ *be a tensor with order* *m* *and dimension* $n\geq2$. *Then all* *Z*-*eigenvalues of* $\mathcal{A}$ *are located in the union of the following sets*:

$$ \sigma(\mathcal{A})\subseteq\mathcal{K}(\mathcal {A})=\bigcup _{i\in N}\mathcal{K}_{i}(\mathcal{A}), $$

*where* $\mathcal{K}_{i}(\mathcal{A})=\{z\in\mathcal{C}:|z|\leq R_{i}(\mathcal{A})\}$ *and* $R_{i}(\mathcal{A})=\sum_{i_{2},\ldots ,i_{m}\in N}|a_{{i}{i_{2}}\ldots{i_{m}}}|$.

By using the partition technique in [16], we present the following notations. Let $\mathcal{A}$ be an *m*th order *n*-dimensional tensor and *S* be a nonempty proper subset of *N*. Set

$$\begin{gathered} \Delta^{N} :=\bigl\{ (i_{2},i_{3}, \ldots,i_{m}): \text{each }i_{j} \in N\text{ for }j = 2,\ldots,m\bigr\} , \\\Delta^{S} :=\bigl\{ (i_{2},i_{3}, \ldots,i_{m}): \text{each } i_{j} \in S\text{ for }j = 2,\ldots,m\bigr\} , \\\overline{\Delta^{S}} = \Delta^{N} \setminus \Delta^{S}. \end{gathered}$$

Then

$$ R_{i}(\mathcal{A})=\sum_{i_{2},\ldots,i_{m} \in N}|a_{{i}{i_{2}}\ldots {i_{m}}}|=R_{i}^{\Delta^{S}}( \mathcal{A})+ R_{i}^{\overline{\Delta ^{S}}}(\mathcal{A}),\quad \forall i \in S, $$

where

$$ R_{i}^{\Delta^{S}}(\mathcal{A}) = \sum _{ ( i_{2},\ldots,i_{m}) \in{\Delta^{S}} } |a_{{i}{i_{2}}\ldots{i_{m}}}|,\qquad R_{i}^{\overline{\Delta^{S}}}( \mathcal {A}) = \sum_{ (i_{2},\ldots,i_{m}) \in\overline{\Delta^{S}} }|a_{{i}{i_{2}}\ldots{i_{m}}}|. $$

### Theorem 1

*Let* $\mathcal{A}$ *be a tensor with order* *m* *and dimension* $n\geq2$ *and* *S* *be a nonempty proper subset of* *N*. *Then all* *Z*-*eigenvalues of* $\mathcal{A}$ *are located in the union of the following sets*:

$$ \sigma(\mathcal{A})\subseteq\mathcal{G}^{S}(\mathcal{A})= \biggl( \bigcup_{i\in S,j\in\bar{S}}\mathcal{G}^{S}_{i,j}( \mathcal{A}) \biggr) \cup \biggl( \bigcup _{i\in\bar{S},j\in S}\mathcal{G}^{\bar{S}}_{i,j}(\mathcal {A}) \biggr), $$

*where*

$$ \begin{gathered} \mathcal{G}_{i,j} ^{S}(\mathcal{A}) = \bigl\{ z\in \mathcal{C}:|z| \bigl(|z|- R_{j}^{\overline{\Delta^{S}}}(\mathcal{A}) \bigr) \leq R_{i} (\mathcal {A})R_{j}^{\Delta^{S}}(\mathcal{A}) \bigr\} , \\ \mathcal{G}_{i,j} ^{\bar{S}}(\mathcal{A}) = \bigl\{ z\in\mathcal {C}:|z| \bigl(|z|-R_{j}^{\overline{\Delta^{ \bar{S}}}}(\mathcal{A}) \bigr) \leq R_{i} (\mathcal{A})R_{j}^{\Delta^{\bar{S}}}(\mathcal{A}) \bigr\} . \end{gathered}$$

### Proof

Let *λ* be a *Z*-eigenvalue of $\mathcal{A}$ with corresponding eigenvector *x*, *i.e*.,

1 $$ \mathcal{A}x^{m-1}=\lambda x,\qquad x^{T}x=1. $$

Let $|x_{t}|={\max\{|x_{i}|: i\in S\}}$, $|x_{s}|= {\max\{ |x_{i}|: i\in\bar{S}\}}$. Then at least one of $|x_{t}|$ and $|x_{s}|$ is nonzero. We next divide the proof into three parts.

(i) If $x_{t}x_{s}\neq0$ and $|x_{s}|\geq|x_{t}|$, then $|x_{s}|={\max\{|x_{i}|: i\in N\}} > 0$. From equality (1), we have

$$\lambda x_{s} = \sum_{ (i_{2},\ldots, i_{m}) \in\Delta^{S} } a_{s i_{2}\ldots i_{m}} x_{i_{2}}\cdots x_{i_{m}}+\sum _{(i_{2},\ldots, i_{m} ) \in\overline{\Delta^{S}} } a_{s i_{2}\ldots i_{m}} x_{i_{2}} \cdots x_{i_{m}}. $$

Noting that $|x_{t}|^{m-1}\leq|x_{t}| \leq1$, $|x_{s}|^{m-1}\leq|x_{s}| \leq 1$ and taking modulus in the above equation, one has

2 $$ \begin{aligned}[b]|\lambda||x_{s}| &\leq\sum _{(i_{2},\ldots, i_{m}) \in\Delta^{S} }|a_{s i_{2}\ldots i_{m}}||x_{i_{2}}| \cdots|x_{i_{m}}| + \sum_{(i_{2},\ldots, i_{m} ) \in\overline{\Delta^{S}} }|a_{s i_{2}\ldots i_{m}}||x_{i_{2}}| \cdots |x_{i_{m}}| \\ &\leq\sum_{(i_{2},\ldots, i_{m}) \in\Delta^{S} }|a_{s i_{2}\ldots i_{m}}||x_{t}|^{m-1} + \sum_{(i_{2},\ldots, i_{m} ) \in\overline{\Delta ^{S}} }|a_{s i_{2}\ldots i_{m}}||x_{s}|^{m-1} \\ &\leq R_{s}^{\Delta^{S}}(\mathcal{A})|x_{t}| + R_{s}^{\overline{\Delta ^{S}}}(\mathcal{A})|x_{s}|. \end{aligned}$$

Dividing both sides by $|x_{s}|$ in (2), we get

3 $$ |\lambda| \leq R_{s}^{\Delta^{S}}(\mathcal{A}) \frac{|x_{t}|}{|x_{s}|} + R_{s}^{\overline{\Delta^{S}}}(\mathcal{A}). $$

On the other hand, by (1), we obtain

$$ |\lambda||x_{t}| \leq\sum_{i_{2},\ldots,i_{m}\in N}|a_{{t}{i_{2}}\ldots {i_{m}}}||x_{i_{2}}| \cdots|x_{i_{m}}|\leq\sum_{i_{2},\ldots,i_{m}\in N}|a_{{t}{i_{2}}\ldots{i_{m}}}||x_{s}|^{m-1}. $$

Dividing both sides by $|x_{t}|$ in the above inequality and from $|x_{s}|^{m-1}\leq|x_{s}|$, one has

4 $$ |\lambda|\leq\sum_{i_{2},\ldots,i_{m}\in N}|a_{{t}{i_{2}}\ldots{i_{m}}}| \frac {|x_{s}|^{m-1}}{|x_{t}|}\leq \sum_{i_{2},\ldots,i_{m}\in N}|a_{{t}{i_{2}}\ldots {i_{m}}}| \frac{|x_{s}|}{|x_{t}|}= R_{t}(\mathcal{A}) \frac{|x_{s}|}{|x_{t}|}. $$

Multiplying (3) by (4), we see

$$ |\lambda| \bigl(|\lambda|- R_{s}^{\overline{\Delta^{S}}}(\mathcal{A}) \bigr) \leq R_{t}(\mathcal{A})R_{s}^{\Delta^{S}}(\mathcal{A}), $$

thus, $\lambda\in\mathcal{G}_{t,s} ^{S}(\mathcal{A})\subseteq \mathcal {G}^{S}(\mathcal{A})$.

(ii) If $x_{t}x_{s}\neq0$ and $|x_{t}|\geq|x_{s}|$, then $|x_{t}|={\max\{|x_{i}|: i\in N \}}$. Similar to the proof of (i), we can get that

$$ |\lambda|- R_{t}^{\overline{\Delta^{\bar{S}}}}(\mathcal{A})\leq R_{t}^{\Delta^{\bar{S}}}( \mathcal{A}) \frac{|x_{s}|}{|x_{t}|} $$

and

$$ |\lambda|\leq R_{s}(\mathcal{A}) \frac{|x_{t}|}{|x_{s}|}, $$

which implies

$$ |\lambda| \bigl(|\lambda|- R_{t}^{\overline{\Delta^{\bar{S}}}}(\mathcal{A}) \bigr) \leq R_{s} (\mathcal{A}) R_{t}^{\Delta^{\bar{S}}}(\mathcal{A}), $$

that is, $\lambda\in \mathcal{G}_{s,t} ^{\bar{S}}(\mathcal {A})\subseteq \mathcal{G}^{S}(\mathcal{A})$.

(iii) If $x_{t}x_{s} = 0$, without loss of generality, let $|x_{t}| = 0$ and $|x_{s}|\neq0$. It follows from (3) that

$$ |\lambda|- R_{s}^{\overline{\Delta^{S}}}(\mathcal{A})\leq 0. $$

For any $i\in S$, we have

$$ |\lambda| \bigl(|\lambda|- R_{s}^{\overline{\Delta^{S}}}(\mathcal{A}) \bigr) \leq0 \leq R_{i}(\mathcal{A})R_{s}^{\Delta^{S}}( \mathcal{A}), $$

that is, $\lambda\in\mathcal{G}_{i,s} ^{S}(\mathcal{A})\subseteq \mathcal{G}^{S}(\mathcal{A})$.

The result follows from (i), (ii) and (iii). □

### Corollary 1

*Let* $\mathcal{A}$ *be a tensor with order* *m* *and dimension* $n\geq2$, *and* *S* *be a nonempty proper subset of* *N*. *Then*

$$ \sigma(\mathcal{A})\subseteq\mathcal{G}^{S}(\mathcal{A})\subseteq \mathcal{K}(\mathcal{A}), $$

*where* $\mathcal{K}(\mathcal{A})$ *is a* *Z*-*eigenvalue inclusion set in Lemma* 1.

### Proof

Let *z* be a point of $\mathcal{K}(\mathcal{A})$. Two cases are discussed as follows:

(i) There exist $t\in S $ and $s \in\bar{S} $ such that $z \in\mathcal {G}_{t,s} ^{S}(\mathcal{A})$, *i.e*.,

5 $$ |z| \bigl(|z|- R_{s}^{\overline{\Delta^{S}}}(\mathcal{A}) \bigr) \leq R_{t} (\mathcal {A})R_{s}^{\Delta^{S}}( \mathcal{A}). $$

If $R_{t} (\mathcal{A})R_{s}^{\Delta^{S}}(\mathcal{A}) = 0 $, then $z = 0$ or $|z|- R_{s}^{\overline{\Delta^{S}}}(\mathcal{A}) \leq0$. Hence, $z\in\mathcal{K}_{t} (\mathcal{A})\cup\mathcal{K}_{s} (\mathcal{A})$. Otherwise, it follows from (5) that

6 $$ \frac{|z|}{R_{t}(\mathcal{A})}\frac{|z|- R_{s}^{\overline{\Delta ^{S}}}(\mathcal{A})}{R_{s}^{\Delta^{S}}(\mathcal{A})}\leq1. $$

Furthermore,

$$ \frac{|z|}{R_{t}(\mathcal{A})}\leq1\quad \text{or} \quad\frac{|z|- R_{s}^{\overline{\Delta^{S}}}(\mathcal{A})}{R_{s}^{\Delta^{S}}(\mathcal {A})}\leq1, $$

that is, $z\in\mathcal{K}_{t} (\mathcal{A})$ or $z\in\mathcal{K}_{s} (\mathcal{A})$. This implies $z\in\mathcal{K}_{t}(\mathcal{A})\cup \mathcal{K}_{s}(\mathcal{A})\subseteq\mathcal{K}(\mathcal{A})$.

(ii) There exist $s \in\bar{S} $ and $t\in S $ such that $z \in\mathcal {G}_{s,t} ^{\bar{S}}(\mathcal{A})$, *i.e*.,

$$ |z| \bigl(|z|-R_{t}^{\overline{\Delta^{ \bar{S}}}}(\mathcal{A}) \bigr) \leq R_{s} (\mathcal{A})R_{t}^{\Delta^{\bar{S}}}(\mathcal{A}), $$

similar to (i), we obtain $z\in\mathcal{K}_{s}(\mathcal {A})\cup\mathcal{K}_{t}(\mathcal{A})\subseteq\mathcal{K}(\mathcal {A})$. So, the result holds. □

Based on an exact characterization of (1), another *S*-type *Z*-eigenvalue localization set involved with a proper subset *S* of *N* is given below.

### Theorem 2

*Let* $\mathcal{A}$ *be a tensor with order* *m* *and dimension* $n\geq2$ *and* *S* *be a nonempty proper subset of* *N*. *Then*

$$ \sigma(\mathcal{A})\subseteq\Omega^{S}(\mathcal {A})= { \biggl( \bigcup_{i\in S,j\in\bar{S}} \bigl(\Omega _{i,j} ^{S}(\mathcal{A})\cup\Phi_{i,j}^{S}( \mathcal{A}) \bigr) \biggr) \cup \biggl( \bigcup _{i\in\bar{S},j\in S} \bigl(\Omega_{i,j} ^{\bar {S}}(\mathcal{A}) \cup\Phi_{i,j}^{\bar{S}}(\mathcal{A}) \bigr) \biggr) }, $$

*where*

$$ \begin{gathered} \Omega_{i,j} ^{S}(\mathcal{A}) = \bigl\{ z\in\mathcal{C}: \bigl(|z|- R_{i}^{\Delta ^{S}}(\mathcal{A}) \bigr) \bigl(|z|-R_{j}^{\overline{\Delta^{S}}}(\mathcal{A}) \bigr) \leq R_{i}^{\overline{\Delta^{S}}}(\mathcal{A})R_{j}^{\Delta^{S}}( \mathcal {A}) \bigr\} , \\ \Omega_{i,j} ^{\bar{S}}(\mathcal{A}) = \bigl\{ z\in\mathcal {C}: \bigl(|z|-R_{i}^{\Delta^{\bar{S}}}(\mathcal{A}) \bigr) \bigl(|z|-R_{j}^{\overline {\Delta^{ \bar{S}}}}( \mathcal{A}) \bigr) \leq R_{i}^{\overline{\Delta^{ \bar {S}}}}(\mathcal{A})R_{j}^{\Delta^{\bar{S}}}( \mathcal{A}) \bigr\} , \\ \Phi_{i,j} ^{S}(\mathcal{A}) = \bigl\{ z\in\mathcal{C}: |z| \leq R_{i}^{\Delta^{S}}(\mathcal{A}),|z|\leq R_{j}^{\overline{\Delta ^{S}}}( \mathcal{A}) \bigr\} , \\ \Phi_{i,j} ^{\bar{S}}(\mathcal{A}) = \bigl\{ z\in\mathcal{C}: |z| \leq R_{i}^{\Delta^{\bar{S}}}(\mathcal{A}), |z|\leq R_{j}^{\overline{\Delta^{ \bar{S}}}}( \mathcal{A}) \bigr\} . \end{gathered}$$

### Proof

Let *λ* be a *Z*-eigenvalue of $\mathcal{A}$ with corresponding eigenvector *x*. Let $|x_{t}|=\max_{i\in S}|x_{i}|$ and $|x_{s}|=\max_{i\in\bar{S}}|x_{i}|$. Similar to the proof of Theorem 1, we also divide the proof into three cases as follows.

(i) If $x_{t}x_{s}\neq0$ and $|x_{s}|\geq|x_{t}|$, then $|x_{s}|={\max\{|x_{i}|: i\in N\}}$. By an exact characterization of (1), one has

$$\begin{aligned} |\lambda||x_{t}|&\leq\sum_{(i_{2},\ldots, i_{m}) \in\Delta^{S} }|a_{t i_{2}\ldots i_{m}}||x_{i_{2}}| \cdots|x_{i_{m}}|+\sum_{(i_{2},\ldots, i_{m} ) \in\overline{\Delta^{S}}}|a_{t i_{2}\ldots i_{m}}||x_{i_{2}}| \cdots|x_{i_{m}}| \\ &\leq R_{t}^{\Delta^{S}}(\mathcal{A}) |x_{t}|^{m-1}+ R_{t}^{\overline {\Delta^{S}}}(\mathcal{A})|x_{s}|^{m-1} \leq R_{t}^{\Delta^{S}}(\mathcal{A}) |x_{t}|+ R_{t}^{\overline{\Delta ^{S}}}(\mathcal{A})|x_{s}|, \end{aligned}$$

since $|x_{t}|^{m-1}\leq|x_{t}| \leq1$, $|x_{s}|^{m-1}\leq|x_{s}| \leq1$ hold. Furthermore,

7 $$ \bigl(|\lambda|-R_{t}^{\Delta^{S}}(\mathcal{A}) \bigr)|x_{t}| \leq R_{t}^{\overline {\Delta^{S}}}( \mathcal{A})|x_{s}|. $$

When $|\lambda| > R_{s}^{\overline{\Delta^{S}}}(\mathcal{A}) $ or $|\lambda|> R_{t}^{\Delta^{S}}(\mathcal{A})$ holds, multiplying (2) by (7), we see

$$ \bigl(|\lambda|-R_{t}^{\Delta^{S}}(\mathcal{A}) \bigr) \bigl(| \lambda|-R_{s}^{\overline {\Delta^{S}}}(\mathcal{A}) \bigr) \leq R_{t}^{\overline{\Delta^{S}}}(\mathcal {A})R_{s}^{\Delta^{S}}( \mathcal{A}). $$

This shows $\lambda\in\Omega_{t,s} ^{S}(\mathcal{A})\subseteq\Omega ^{S}(\mathcal{A})$. Otherwise, when $|\lambda|\leq R_{s}^{\overline{\Delta^{S}}}(\mathcal{A}) $ and $|\lambda|\leq R_{t}^{\Delta^{S}}(\mathcal{A})$ hold, one has $\lambda\in\Phi_{t,s} ^{S}(\mathcal{A})\subseteq \Omega^{S}(\mathcal{A})$.

(ii) If $x_{t}x_{s}\neq0$ and $|x_{t}|\geq|x_{s}|$, then $|x_{t}|={\max\{|x_{i}|: i\in N \}}$. Similarly, by equality (1), we get

$$ \bigl(|\lambda|-R_{t}^{\overline{\Delta^{\bar{S}}}}(\mathcal{A}) \bigr)|x_{t}| \leq R_{t}^{\Delta^{\bar{S}}}( \mathcal{A})|x_{s}| $$

and

$$ \bigl(|\lambda|- R_{s}^{\Delta^{\bar{S}}}(\mathcal{A}) \bigr)|x_{s}| \leq R_{s}^{\overline{\Delta^{\bar{S}}}}( \mathcal{A})|x_{t}|. $$

When $|\lambda|- R_{s}^{\Delta^{\bar{S}}}(\mathcal{A})> 0 $ or $|\lambda |-R_{t}^{\overline{\Delta^{\bar{S}}}}(\mathcal{A})>0$ holds, we obtain

$$ \bigl(|\lambda|- R_{s}^{\Delta^{\bar{S}}}(\mathcal{A}) \bigr) \bigl(| \lambda|- R_{t}^{\overline{\Delta^{\bar{S}}}}(\mathcal{A}) \bigr) \leq R_{t}^{\Delta^{\bar {S}}}(\mathcal{A})R_{s}^{\overline{\Delta^{\bar{S}}}}( \mathcal{A}), $$

which implies $\lambda\in\Omega_{s,t} ^{\bar{S}}(\mathcal{A})\subseteq \Omega^{S}(\mathcal{A})$. When $|\lambda|- R_{s}^{\Delta^{\bar{S}}}(\mathcal{A}) \leq0 $ and $|\lambda|-R_{t}^{\overline{\Delta^{\bar{S}}}}(\mathcal{A})\leq0$ hold, one has $\lambda\in\Phi_{s,t} ^{\bar{S}}(\mathcal{A})\subseteq \Omega ^{S}(\mathcal{A})$.

(iii) If $|x_{t}||x_{s}| = 0$, we could assume that $|x_{s}| = 0$ and $|x_{t}|\neq0$. It follows from (7) that

$$ |\lambda|-R_{t}^{\Delta^{S}}(\mathcal{A})\leq0. $$

For any $j\in \bar{S}$, when $|\lambda|-R_{j}^{\overline{\Delta ^{S}}}(\mathcal{A})> 0$ holds, we get

$$ \bigl(|\lambda|-R_{t}^{\Delta^{S}}(\mathcal{A}) \bigr) \bigl(| \lambda|- R_{j}^{\overline{\Delta^{S}}}(\mathcal{A}) \bigr)\leq R_{t}^{\overline{\Delta ^{S}}}(\mathcal{A})R_{j}^{\Delta^{S}}( \mathcal{A}), $$

that is, $\lambda\in\Omega_{t,j} ^{S}(\mathcal{A})\subseteq\Omega ^{S}(\mathcal{A})$; otherwise, when $|\lambda|-R_{j}^{\overline{\Delta ^{S}}}(\mathcal{A})\leq0$ holds, $\lambda\in\Phi_{t,j} ^{S}(\mathcal {A})\subseteq \Omega^{S}(\mathcal{A})$. It follows from (i), (ii) and (iii) that the results hold. □

### Corollary 2

*Let* $\mathcal{A}$ *be a tensor with order* *m* *and dimension* $n\geq2$.

(I) *If there exists* $S\subseteq N$ *such that*
  (i) *for all* $i \in S$, $j\in\bar{S}$, $R_{j}^{\overline{\Delta ^{S}}}(\mathcal{A})\leq|z|\leq R_{i}(\mathcal{A})$ *and* $R_{i}^{\overline {\Delta^{S}}}(\mathcal{A})R_{j}^{\Delta^{S}}(\mathcal{A})> 0$ *hold*;
  (ii) *for all* $i \in\bar{S}$, $j\in S$, $R_{j}^{\overline {\Delta^{\bar{S}}}}(\mathcal{A}) \leq|z|\leq R_{i}(\mathcal{A})$ *and* $R_{i}^{\overline{\Delta^{\bar{S}}}}(\mathcal{A}) R_{j}^{\Delta^{\bar {S}}}(\mathcal{A}) > 0$ *hold*, *then*
    $$ \mathcal{G}^{S}(\mathcal{A})\subseteq\Omega^{S}( \mathcal{A}). $$
(II) *If there exists* $S\subseteq N$ *such that*
  (i) *for all* $i \in S$, $j\in\bar{S}$, $|z|\leq\min\{ R_{i}^{\Delta^{S}}(\mathcal{A}), R_{j}^{\overline{\Delta^{S}}}(\mathcal {A})\}$ *holds*; *or* $|z|\geq\max\{R_{i}(\mathcal{A}), R_{j}^{\overline {\Delta^{S}}}(\mathcal{A})\}$ *and* $R_{i}^{\overline{\Delta^{S}}}(\mathcal{A})R_{j}^{\Delta^{S}}(\mathcal {A})> 0$ *are satisfied*;
  (ii) *for all* $i \in\bar{S}$, $j\in S$, $|z|\leq\min\{ R_{i}^{\Delta^{\bar{S}}}(\mathcal{A}), R_{j}^{\overline{\Delta^{ \bar {S}}}}(\mathcal{A})\}$ *holds*; *or* $|z|\geq\max\{ R_{i}(\mathcal{A}), R_{j}^{\overline{\Delta^{\bar{S}}}}(\mathcal{A})\}$ *and* $R_{i}^{\overline {\Delta^{\bar{S}}}}(\mathcal{A})R_{j}^{\Delta^{\bar{S}}}(\mathcal{A}) > 0$ *are satisfied*, *then*
    $$ \Omega^{S}(\mathcal{A})\subseteq\mathcal{G}^{S}( \mathcal{A}). $$

### Proof

(I) Let $z\in\mathcal{G}^{S}(\mathcal{A})$, then $z\in\mathcal{G}^{S}_{i,j}(\mathcal{A})$ or $z\in\mathcal{G}^{\bar {S}}_{i,j}(\mathcal{A})$. We divide the proof into two parts.

(i) Suppose that $z\in\mathcal{G}^{S}_{i,j}(\mathcal{A})$, then there exist $t\in S $ and $s \in\bar{S} $ such that $z\in\mathcal {G}_{t,s} ^{S}(\mathcal{A})$.

If $R_{t} (\mathcal{A}) = 0 $, then $R_{t}^{\Delta^{S}}(\mathcal{A})= R_{t}^{\overline{\Delta^{S}}}(\mathcal{A})=0$, we have $z = 0$ or $|z|- R_{s}^{\overline{\Delta^{S}}}(\mathcal{A}) \leq0$. Hence, $z\in\Omega _{t,s} ^{S}(\mathcal{A})$.

If $R_{t} (\mathcal{A})R_{s}^{\Delta^{S}}(\mathcal{A}) >0 $, by (6), we have

$$ \frac{|z|}{R_{t}(\mathcal{A})}\leq1 \quad\text{or} \quad\frac{|z|- R_{s}^{\overline{\Delta^{S}}}(\mathcal{A})}{R_{s}^{\Delta^{S}}(\mathcal {A})}\leq1. $$

When $\frac{|z|- R_{s}^{\overline{\Delta^{S}}}(\mathcal{A})}{R_{s}^{\Delta ^{S}}(\mathcal{A})}\geq0$ and $\frac{|z|}{R_{t}(\mathcal{A})}\leq1 $, letting $a=|z|$, $b=R_{t}^{\Delta^{S}}(\mathcal{A})$, $c=0$, $d=R_{t}^{\overline{\Delta^{S}}}(\mathcal{A})>0$, from Lemma 5 in [16] and (6), we get

$$ \frac{|z|-R_{t}^{\Delta^{S}}(\mathcal {A})}{R_{t}^{\overline{\Delta^{S}}}(\mathcal{A})}\frac{|z|- R_{s}^{\overline{\Delta^{S}}}(\mathcal{A})}{R_{s}^{\Delta^{S}}(\mathcal {A})}\leq\frac{|z|}{R_{t}(\mathcal{A})}\frac{|z|- R_{s}^{\overline{\Delta ^{S}}}(\mathcal{A})}{R_{s}^{\Delta^{S}}(\mathcal{A})}\leq1. $$

Furthermore,

$$ \bigl(|z|-R_{t}^{\Delta^{S}}(\mathcal{A}) \bigr) \bigl(|z|-R_{s}^{\overline {\Delta^{S}}}(\mathcal{A}) \bigr) \leq R_{t}^{\overline{\Delta^{S}}}(\mathcal {A})R_{s}^{\Delta^{S}}( \mathcal{A}), $$

which implies $z\in\Omega_{t,s} ^{S}(\mathcal{A})$. So,

$$ z\in\mathcal{G}^{S}_{t,s}(\mathcal{A}) \subseteq\Omega _{t,s} ^{S}(\mathcal{A}) \quad\mbox{and} \quad\mathcal{G}^{S}( \mathcal {A})\subseteq\Omega^{S}(\mathcal{A}). $$

(ii) Suppose that $z\in\mathcal{G}^{\bar{S}}(\mathcal {A})$, then there exist $s \in\bar{S} $ and $t\in S $ such that $z \in\mathcal {G}_{s,t} ^{\bar{S}}(\mathcal{A})$. Similar to the proof of (i), the conclusion holds.

(II) Let $z\in\Omega^{S}(\mathcal{A})$, then $z\in\bigcup_{i\in S,j\in\bar{S}}\Omega_{i,j} ^{S}(\mathcal {A})\cup\Phi_{i,j}^{S}(\mathcal{A})$ or $z\in\bigcup _{i\in \bar{S},j\in S}\Omega_{i,j} ^{\bar{S}}(\mathcal{A})\cup\Phi _{i,j}^{\bar{S}}(\mathcal{A})$. We also divide the proof into two parts.

(i) Suppose that $z\in\bigcup_{i\in S,j\in\bar {S}}\Omega_{i,j} ^{S}(\mathcal{A})\cup\Phi_{i,j}^{S}(\mathcal{A})$, then there exist $t\in S $ and $s \in\bar{S} $ such that $z\in\Omega _{t,s} ^{S}(\mathcal{A})$ or $z\in\Phi_{t,s} ^{S}(\mathcal{A})$.

If $z\in\Phi_{t,s} ^{S}(\mathcal{A})$, that is, $|z| \leq R_{t}^{\Delta ^{S}}(\mathcal{A})$ and $|z|\leq R_{s}^{\overline{\Delta^{S}}}(\mathcal {A})$, then it is easy to get that $\Omega^{S}(\mathcal{A})\subseteq \mathcal{G}^{S}(\mathcal{A})$.

If $z\in\Omega_{t,s} ^{S}(\mathcal{A})$, that is,

8 $$ \bigl(|z|-R_{t}^{\Delta^{S}}(\mathcal{A}) \bigr) \bigl(|z|-R_{s}^{\overline{\Delta ^{S}}}(\mathcal{A}) \bigr) \leq R_{t}^{\overline{\Delta^{S}}}(\mathcal {A})R_{s}^{\Delta^{S}}( \mathcal{A}). $$

We assume $R_{t}^{\overline{\Delta^{S}}}(\mathcal{A})R_{s}^{\Delta ^{S}}(\mathcal{A})>0 $, it follows from (8) that

9 $$ \frac{|z|-R_{t}^{\Delta^{S}}(\mathcal{A})}{R_{t}^{\overline{\Delta ^{S}}}(\mathcal{A})}\frac{|z|- R_{s}^{\overline{\Delta^{S}}}(\mathcal {A})}{R_{s}^{\Delta^{S}}(\mathcal{A})}\leq1. $$

When $\frac{|z|- R_{s}^{\overline{\Delta^{S}}}(\mathcal{A})}{R_{s}^{\Delta ^{S}}(\mathcal{A})}\geq0$ and $\frac{|z|}{R_{t}(\mathcal{A})}\geq1 $, letting $a=|z|$, $b=R_{t}^{\Delta^{S}}(\mathcal{A})$, $c=0$, $d=R_{t}^{\overline{\Delta^{S}}}(\mathcal{A})>0$, from Lemma 5 in [16] and (9), we obtain

$$ \frac{|z|}{R_{t}(\mathcal{A})}\frac{|z|- R_{s}^{\overline {\Delta^{S}}}(\mathcal{A})}{R_{s}^{\Delta^{S}}(\mathcal{A})}\leq\frac {|z|-R_{t}^{\Delta^{S}}(\mathcal{A})}{R_{t}^{\overline{\Delta ^{S}}}(\mathcal{A})}\frac{|z|- R_{s}^{\overline{\Delta^{S}}}(\mathcal {A})}{R_{s}^{\Delta^{S}}(\mathcal{A})} \leq1. $$

Moreover,

$$ |z| \bigl(|z|-R_{s}^{\overline{\Delta^{S}}}(\mathcal{A}) \bigr) \leq R_{t}(\mathcal{A})R_{s}^{\Delta^{S}}(\mathcal{A}), $$

which implies $z\in\mathcal{G}_{t,s} ^{S}(\mathcal{A})$. Hence,

$$ z\in\Omega_{t,s} ^{S}(\mathcal{A}) \subseteq \mathcal {G}^{S}_{t,s}(\mathcal{A}) \quad \mbox{and} \quad\Omega^{S}( \mathcal{A})\subseteq \mathcal{G}^{S}(\mathcal{A}). $$

(ii) Suppose that $z\in\bigcup_{i\in\bar {S},j\in S}{ (\Omega_{i,j} ^{\bar{S}}(\mathcal {A})\cup\Phi_{i,j}^{\bar{S}}(\mathcal{A}) )}$. Similar to the proof of (i), we arrive at the result. □

Owing to the uncertainty of *S*, we cannot compare $\mathcal {G}^{S}(\mathcal{A})$ with $\Omega^{S}(\mathcal{A})$ theoretically without the conditions of Corollary 2. Example 1 shows that they are different, since $\mathcal{G}^{S}_{i,j}$ ($\mathcal{A})(\mathcal{G}^{\bar {S}}_{i,j}(\mathcal{A})$) and $\Omega_{i,j} ^{S}$ ($\mathcal{A})(\Omega _{i,j} ^{\bar{S}}(\mathcal{A}) $) do not include each other.

### Example 1

Let $\mathcal{A}=(a_{ijk})\in\mathcal{R}^{[3,3]}$ be a tensor with elements defined as follows:

$$ a_{ijk}=\left \{ \textstyle\begin{array}{ll} a_{111}=1;\qquad a_{121}=-1; \qquad a_{122}=1;\qquad a_{133}=-1;\\ a_{211}=-1; \qquad a_{213}=1;\qquad a_{221}=2;\qquad a_{233}=-1;\\ a_{311}=3; \qquad a_{322}=-1;\qquad a_{332}=-1;\qquad a_{333}=1;\\ a_{ijk}=0, \quad \text{otherwise}. \end{array}\displaystyle \right . $$

According to Lemma 1, we have

$$ \mathcal{K}(\mathcal{A})=\bigcup_{i\in N}\mathcal {K}_{i}(\mathcal{A})=\bigl\{ \lambda\in C: |\lambda|\leq6\bigr\} . $$

Let $S=\{{1}\}$. Obviously, $\bar{S}=\{{2,3}\}$. From Theorem 1, one has

$$ \sigma(\mathcal{A})\subseteq\mathcal{G}^{S}(\mathcal {A})= \biggl\{ \lambda\in C: |\lambda|\leq\frac{3+\sqrt{57}}{2} \biggr\} , $$

where

$$\begin{gathered}\mathcal{G}^{S}_{1,2}(\mathcal{A})=\bigl\{ \lambda\in C: |\lambda| \leq 2+2\sqrt{2}\bigr\} , \qquad\mathcal{G}^{S}_{1,3}(\mathcal{A})= \biggl\{ \lambda\in C: |\lambda|\leq\frac{3+\sqrt{57}}{2}\biggr\} , \\ \mathcal{G}^{\bar{S}}_{2,1}(\mathcal{A})=\bigl\{ \lambda\in C: |\lambda |\leq1+\sqrt{11}\bigr\} , \qquad \mathcal{G}^{\bar{S}}_{3,1}( \mathcal{A})=\bigl\{ \lambda\in C: |\lambda|\leq1+\sqrt{13}\bigr\} .\end{gathered} $$

And it follows from Theorem 2 that

$$ \sigma(\mathcal{A})\subseteq\Omega^{S}(\mathcal{A})=\bigl\{ \lambda\in C: | \lambda|\leq 2+\sqrt{10}\bigr\} , $$

where

$$\begin{gathered}\Omega^{S}_{1,2}(\mathcal{A})=\biggl\{ \lambda\in C: |\lambda| \leq\frac {5+\sqrt{21}}{2}\biggr\} , \qquad \Omega^{S}_{1,3}( \mathcal{A})=\bigl\{ \lambda\in C: |\lambda|\leq2+\sqrt{10}\bigr\} , \\ \Omega^{\bar{S}}_{2,1}(\mathcal{A})=\biggl\{ \lambda\in C: |\lambda|\leq \frac{3+\sqrt{33}}{2}\biggr\} , \qquad \Omega^{\bar{S}}_{3,1}( \mathcal{A})=\bigl\{ \lambda\in C: |\lambda|\leq5\bigr\} .\end{gathered} $$

## Bounds on the largest *Z*-eigenvalue of weakly symmetric nonnegative tensors

In this section, by Theorem 1 and Theorem 2, we give new sharp upper bounds for weakly symmetric nonnegative tensors, which improve the results of [1, 8, 10–12, 14] in a sense. We start this section with some fundamental results of nonnegative tensors [8].

### Lemma 2

Theorem 3.11 of [8]

*Assume that* $\mathcal{A}$ *is a weakly symmetric nonnegative tensor*. *Then* $\rho (\mathcal{A})=\lambda^{*}$, *where* $\lambda^{*}$ *denotes the largest* *Z*-*eigenvalue*.

### Theorem 3

*Suppose that an* *m*-*order* *n*-*dimensional nonnegative tensor* $\mathcal{A}$ *is weakly symmetric and* *S* *is a nonempty proper subset of* *N*. *Then*

$$ \rho(\mathcal{A})\leq u_{S}=\max \bigl\{ u^{S}, u^{\bar{S}} \bigr\} , $$

*where*

$$\begin{gathered} u^{S}=\max_{i\in S, j\in\bar{S}}\frac{1}{2} \Bigl\{ R_{j}^{\overline{\Delta^{S}}}(\mathcal{A})+\sqrt{ \bigl(R_{j}^{\overline{\Delta ^{S}}}( \mathcal{A}) \bigr)^{2}+4R_{i}( \mathcal{A})R_{j}^{\Delta^{S}} (\mathcal {A})} \Bigr\} , \\ u^{\bar{S}}= \max_{i\in\bar{S}, j\in S}\frac{1}{2} \Bigl\{ R_{j}^{\overline {\Delta^{\bar{S}}}}(\mathcal{A})+\sqrt{ \bigl(R_{j}^{\overline{\Delta^{\bar {S}}}}(\mathcal{A}) \bigr)^{2}+4R_{i} (\mathcal{A})R_{j}^{\Delta^{\bar {S}}}(\mathcal{A})} \Bigr\} . \end{gathered}$$

### Proof

According to Lemma 2, we assume that $\rho(\mathcal{A})= \lambda ^{*}$ is the largest *Z*-eigenvalue of $\mathcal{A}$. From Theorem 1, we get

$$ \rho(\mathcal{A}) \in\bigcup_{i\in S,j\in\bar {S}} \mathcal{G}^{S}_{i,j}(\mathcal{A}) $$

or

$$ \rho(\mathcal{A}) \in\bigcup_{i\in\bar{S},j\in S} \mathcal{G}^{\bar{S}}_{i,j}(\mathcal{A}). $$

For the case that $\rho(\mathcal{A}) \in\bigcup_{i\in S,j\in \bar{S}}\mathcal{G}^{S}_{i,j}(\mathcal{A})$, there exist $t \in S $, $s \in \bar{S}$ such that

10 $$ \bigl(\rho(\mathcal{A})- R_{s}^{\overline{\Delta^{S}}}( \mathcal{A}) \bigr)\rho (\mathcal{A}) \leq R_{t} (\mathcal{A})R_{s}^{\Delta^{S}}( \mathcal{A}). $$

Solving $\rho(\mathcal{A})$ in inequality (10), we obtain

11 $$ \rho(\mathcal{A})\leq\frac{1}{2} \Bigl\{ R_{s}^{\overline{\Delta^{S}}}( \mathcal {A})+\sqrt{ \bigl(R_{s}^{\overline{\Delta^{S}}}(\mathcal{A}) \bigr)^{2}+4R_{t}(\mathcal {A})R_{s}^{\Delta^{S}} (\mathcal{A})} \Bigr\} . $$

Furthermore,

12 $$ \rho(\mathcal{A})\leq\max_{i\in S, j\in\bar{S}} \frac{1}{2} \Bigl\{ R_{j}^{\overline{\Delta^{S}}}(\mathcal{A})+\sqrt { \bigl(R_{j}^{\overline{\Delta ^{S}}}(\mathcal{A}) \bigr)^{2}+4R_{i}( \mathcal{A})R_{j}^{\Delta^{S}} (\mathcal {A})} \Bigr\} . $$

For another case that $\rho(\mathcal{A}) \in\bigcup_{i\in\bar {S},j\in S}\mathcal{G}^{\bar{S}}_{i,j}(\mathcal{A})$, we also get

13 $$ \rho(\mathcal{A})\leq\max_{i\in\bar{S}, j\in S} \frac{1}{2} \Bigl\{ R_{j}^{\overline{\Delta^{\bar{S}}}}(\mathcal{A})+\sqrt { \bigl(R_{j}^{\overline {\Delta^{\bar{S}}}}(\mathcal{A}) \bigr)^{2}+4R_{i} (\mathcal{A})R_{j}^{\Delta ^{\bar{S}}}( \mathcal{A})} \Bigr\} . $$

It follows from (12) and (13) that the upper bound holds. □

On the basis of Theorem 2, we obtain another sharp bound of the largest *Z*-eigenvalue for a weakly symmetric nonnegative tensor.

### Theorem 4

*Suppose that an* *m*-*order* *n*-*dimensional nonnegative tensor* $\mathcal {A}$ *is weakly symmetric and* *S* *is a nonempty proper subset of* *N*. *Then*

$$ \rho(\mathcal{A})\leq v_{S}=\max \Bigl\{ \max_{i\in S, j\in\bar{S}} \bigl\{ \hat{v}^{S}, \tilde{v}^{S} \bigr\} , \max _{i\in\bar{S}, j\in S} \bigl\{ \hat{v}^{\bar{S}},\tilde{v}^{\bar{S}} \bigr\} \Bigr\} , $$

*where*

$$\begin{gathered} \hat{v}^{S}=\min_{i\in S, j\in\bar{S}} \bigl\{ R_{i}^{\Delta^{S}}( \mathcal {A}),R_{j}^{\overline{\Delta^{S}}}(\mathcal{A}) \bigr\} , \\ \tilde{v}^{S}= \frac{1}{2} \Bigl\{ R_{i}^{\Delta^{S}}( \mathcal {A})+R_{j}^{\overline{\Delta^{S}}}(\mathcal{A})+\sqrt{ \bigl(R_{i}^{\Delta ^{S}}(\mathcal{A})-R_{j}^{\overline{\Delta^{S}}}( \mathcal {A}) \bigr)^{2}+4R_{i}^{\overline{\Delta^{S}}}( \mathcal{A})R_{j}^{\Delta ^{S}}(\mathcal{A})} \Bigr\} . \end{gathered}$$

### Proof

Similar to the proof of Theorem 3, according to Lemma 2 and Theorem 2, the conclusion holds. □

### Remark 1

For a weakly symmetric nonnegative tensor $\mathcal{A}$, as shown in the proofs of Theorem 3 and Theorem 4, it is not hard to obtain that

$$ u_{S} \leq\max_{i\in N} R_{i}(\mathcal{A}) \quad\text{and} \quad v_{S} \leq\max_{i\in N} R_{i}( \mathcal{A}). $$

Next, we take the following example to show the efficiency of our new upper bounds.

### Example 2

[12]

Consider 3 order 3 dimensional tensor $\mathcal{A}=(a_{ijk})$ defined by

$$ a_{ijk}=\left \{ \textstyle\begin{array}{ll} a_{111}=\frac{1}{2};\qquad a_{222}=1; \qquad a_{333}=3;\\ a_{ijk}=\frac{1}{3}, \quad \text{otherwise}. \end{array}\displaystyle \right . $$

By computation, we get $(\rho(\mathcal{A}),x)=(3.1970,(0.1927, 0.1990, 0.9609))$.

From Proposition 3.3 of [8], we have

$$ \rho(\mathcal{A})\leq9.8150. $$

From Corollary 4.5 of [10], we have

$$ \rho(\mathcal{A})\leq5.6667. $$

From Theorem 2.7 of [11], we have

$$ \rho(\mathcal{A})\leq5.6079. $$

From Theorem 7 of [12], we have

$$ \rho(\mathcal{A})\leq5.3654. $$

From Theorem 3.3 of [14], we have

$$ \rho(\mathcal{A})\leq5.5494. $$

From Theorem 4.7 of [1], we have

$$ \rho(\mathcal{A})\leq5.2624. $$

Let $S=\{{3}\}$, then $\bar{S}=\{{1,2}\}$. By Theorem 3, we obtain

$$ \rho(\mathcal{A})\leq5.2624; $$

according to Theorem 4, we obtain

$$ \rho(\mathcal{A})\leq5.0596. $$

## Conclusions

In this paper, we consider the *Z*-eigenvalue for general tensors and obtain two new *S*-type *Z*-eigenvalue inclusion sets. According to the above results, we present upper bounds on the spectral radius of weakly symmetric nonnegative tensors and show that the results are sharper than the upper bounds provided by [1, 8, 10–12, 14] in Example 2.
